# Data on correlation between CT-derived and MRI-derived myocardial extracellular volume

**DOI:** 10.1016/j.dib.2016.03.073

**Published:** 2016-03-28

**Authors:** Yoshie Kurita, Kakuya Kitagawa, Yusuke Kurobe, Shiro Nakamori, Hiroshi Nakajima, Kaoru Dohi, Masaaki Ito, Hajime Sakuma

**Affiliations:** a Department of Radiology and Mie University Hospital, Tsu, Japan; b Department of Cardiology, Mie University Hospital, Tsu, Japan

## Abstract

This article describes data related to a research article titled “Estimation of myocardial extracellular volume fraction with cardiac CT in subjects without clinical coronary artery disease: A feasibility study”, Kurita et al. (in press) [1]. Myocardial extracellular volume fraction (ECV) is an imaging biomarker that can elevate in various heart diseases. This article describes correlation between CT-derived and MRI-derived ECV in 24 myocardial segments in 8 patients. CT-derived ECV was obtained from pre-contrast and delayed-phase images acquired by using dual-source CT system. MRI-derived ECV was obtained by using modified Look-Locker inversion recovery sequence implemented on a 3 T MRI system.

**Specifications Table**

**Table:** 

Subject area	*Radiology*
More specific subject area	*Multi-detector computed tomography*
Type of data	*Figures*
How data was acquired	*Dual-source CT system (Definition Flash, Siemens Healthcare, Forchheim, Germany) and post processing workstation (Ziostation; Ziosoftware, Tokyo, Japan and Vitrea CT myocardial analysis, Vitral Images, Minnetonka, MN). 3 T MRI system (Ingenia 3 T with dSTorso coils, Philips Medical Systems, Best, The Netherlands) and post processing workstation (cvi42, Circle, Calgary, Canada).*
Data format	*Analyzed*
Experimental factors	*Extracellular volume fraction of left ventricular myocardium was measured both by CT and by MRI.*
Experimental features	*Extracellular volume fraction measured by CT correlated with that measured by MRI.*
Data source location	*Tsu, Mie, Japan*
Data accessibility	*Data is within this article*

**Value of the data**

• Provide the reliability data of CT measurement of ECV by employing MRI T1 mapping as a gold standard.
• This data is important because validation to the accuracy of ECV measured by CT is limited in the existing literature.
• This data may stimulate future research investigating ECV in patients with various heart diseases using CT, particularly when MRI T1 mapping is contraindicated or not available.

## 1 Data

We present the result of direct comparison of extracellular volume fraction (ECV) determined by CT and that by MRI T1 mapping. The data were obtained from 24 segments in 8 patients; 2 patients with old myocardial infarction, 2 with hypertrophic cardiomyopathy, 2 without late gadolinium enhancement, 1 with dilated cardiomyopathy, and 1 with primary aldosteronism.

## 2 Experimental design, materials and methods

We have demonstrated that CT measurement of myocardial ECV has high inter- and intra-observer reproducibility [1]. In order to investigate agreement of the ECV measured by CT with that measured by MRI T1 mapping, on which only one publication is available [2], we retrospectively identified 8 patients who underwent both CT and MRI T1 mapping from 187 patients who underwent comprehensive cardiac CT study between March in 2012 and February in 2014.

**Fig. 1:**
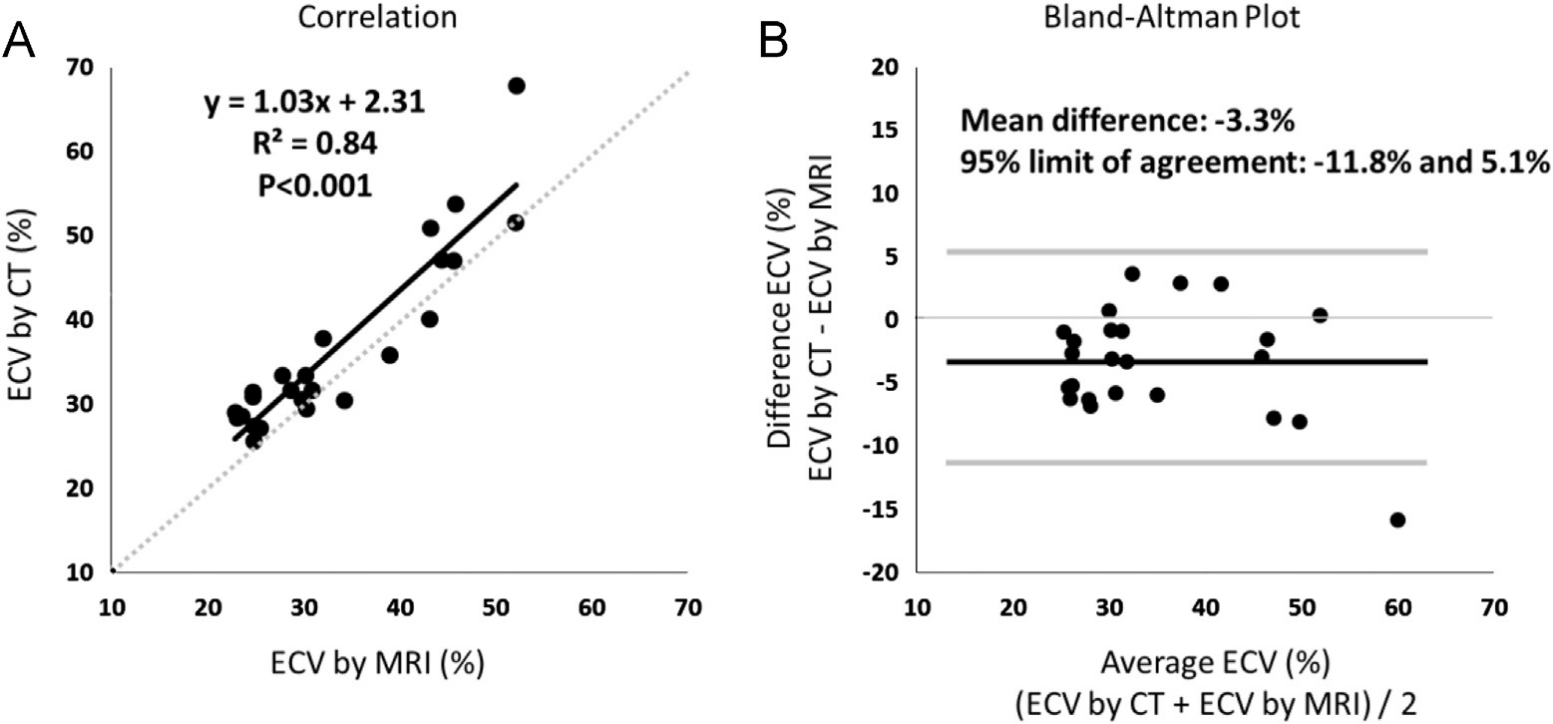
Correlation (A) and Bland–Altman plot (B) between extracellular volume measured by CT and that by MRI. There was a significant correlation (*R*^2^=0.84) with a mean difference of −3.3%.

Pre- and post-contrast T1 mapping of 3 left ventricular short axis slices was performed by using 3-3-5 modified Look-Locker inversion recovery sequence (MOLLI) with field of view of 300×330 mm, acquisition matrix of 176×141 mm, repetition time of 2.6 ms, echo time of 1.1 ms, flip angle of 35 degrees, slice thickness of 10 mm and SENSE factor of 2. Then ECV was determined at representative 3 locations with or without enhancement by placing regions of interest (ROI) [2]. CT measurement of ECV in the 24 locations (3 locations × 8 patients) were performed as described in our original research article [1]. Graph of correlation and Bland–Altman Plot between ECV by CT and ECV by MRI were presented (Fig. 1). All statistical analyses were performed using the GraphPad PRISM 6 (GraphPad Software, Inc, La Jolla, CA).
